# Blood-based biomarkers of inflammation in amyotrophic lateral sclerosis

**DOI:** 10.1186/s13024-022-00515-1

**Published:** 2022-01-24

**Authors:** Kim A. Staats, David R. Borchelt, Malú Gámez Tansey, James Wymer

**Affiliations:** 1 Staats Life Sciences Consulting, LLC, Los Angeles, CA USA; 2 Department of Neuroscience, University of Florida College of Medicine, McKnight Brain Institute, Center for Translational Research in Neurodegenerative Disease, University of Florida, Gainesville, Florida USA; 3 Department of Neuroscience and Center for Translational Research in Neurodegenerative Disease at The University of Florida College of Medicine, Gainesville, Florida USA; 4 Department of Neurology, University of Florida College of Medicine, Gainesville, Florida USA

**Keywords:** Amyotrophic lateral sclerosis, Biomarker, Inflammation, Cytokines

## Abstract

Amyotrophic Lateral Sclerosis (ALS) is a devastating neurodegenerative disease in which many processes are detected including (neuro)inflammation. Many drugs have been tested for ALS in clinical trials but most have failed to reach their primary endpoints. The development and inclusion of different types of biomarkers in diagnosis and clinical trials can assist in determining target engagement of a drug, in distinguishing between ALS and other diseases, and in predicting disease progression rate, drug responsiveness, or an adverse event. Ideally, among other characteristics, a biomarker in ALS correlates highly with a disease process in the central nervous system or with disease progression and is conveniently obtained in a peripheral tissue. Here, we describe the state of biomarkers of inflammation in ALS by focusing on peripherally detectable and cellular responses from blood cells, and provide new (combinatorial) directions for exploration that are now feasible due to technological advancements.

## Background

The efforts to develop an effective treatment in Amyotrophic Lateral Sclerosis (ALS), have resulted in over 40 failed drugs in clinical trials for ALS, testing a broad range of targets [1]. In most of these clinical trials, it is difficult to ascertain the reason for the failure. The drug may have failed to engage its target, had efficacy in a subgroup of patients, or the therapeutic target was not relevant to disease progression at all, or within the clinical trial duration. To promote more informative clinical trials, the Food and Drug Administration (FDA) has included the co-development of biomarkers in their ALS drug development guidance for industry (finalized September 2019), and defines a biomarker as ‘a defined characteristic that is measured as an indicator of normal biological processes, pathogenic processes, or responses to an exposure or intervention, including therapeutic interventions’ [2]. In this manuscript, we describe the state of biomarkers of inflammation in ALS by focusing on peripherally detectable and cellular responses from blood cells, per FDA defined subtype of biomarker. In addition, we provide new (combinatorial) directions for exploration that are now feasible due to technological advancements.

### Definitions of biomarkers

To harmonize used terminology, the Food and Drugs Administration (FDA) has defined a biomarker and seven subtypes of biomarkers through the Biomarker EndpointS and other Tools (BEST) resource [2], as the following.

#### Biomarker

A defined characteristic that is measured as an indicator of normal biological processes, pathogenic processes, or responses to an exposure or intervention, including therapeutic interventions. Molecular, histologic, radiographic, or physiologic characteristics are types of biomarkers. A biomarker is not an assessment of how a patient feels, functions, or survives.

#### Diagnostic biomarker

A biomarker used to detect or confirm presence of a disease or condition of interest or to identify individuals with a subtype of the disease.

#### Monitoring biomarker

A biomarker measured serially for assessing status of a disease or medical condition or for evidence of exposure to (or effect of) a medical product or an environmental agent.

*Pharmacodynamic/Response biomarker*: A biomarker used to show that a biological response has occurred in an individual who has been exposed to a medical product or an environmental agent.

#### Predictive biomarker

A biomarker used to identify individuals who are more likely than similar individuals without the biomarker to experience a favorable or unfavorable effect from exposure to a medical product or an environmental agent.

#### Prognostic biomarker

A biomarker used to identify likelihood of a clinical event, disease recurrence or progression in patients who have the disease or medical condition of interest.

#### Safety biomarker

A biomarker measured before or after an exposure to a medical product or an environmental agent to indicate the likelihood, presence, or extent of toxicity as an adverse effect.

#### Susceptibility/risk biomarker

A biomarker that indicates the potential for developing a disease or medical condition in an individual who does not currently have clinically apparent disease or the medical condition.

### Search strategy and selection criteria

References for this review were identified by searches of PubMed up to February 2021, and references from relevant articles. The search terms “ALS + blood + inflammation” and “Amyotrophic + blood + inflammation” were used, with recommended additions by reviewers. Phase 3 studies in Table 3 were identified by assessing clinicaltrials.gov for entries up to February 2021 that matched the search terms “ALS” (condition or disease) and “phase 3” (other terms). During filtering, studies with a recruitment status of “suspended”, “terminated”, or “withdrawn” were removed from assessment. The remaining studies were subsequently analyzed for the medicinal product’s main target and suitability for this review. There were no language restrictions. The final reference list was generated on the basis of relevance to the topics covered in this review

## Introduction

ALS is a devastating neurodegenerative disease, characterized by a progressive loss of motor neurons, paralysis, and inflammation with an average survival of 3–5 years after diagnosis. Although ~ 5–10% of cases are familial, and ~ 70% of the familial cases can be explained by identified gene mutations, e.g. the *C9orf72* repeat expansion [7, 8], most cases are of a sporadic nature whereby no other family member is known with the disease [9]. Only two drugs have received market approval in the USA, Riluzole and Edaravone, and one in the EU, Riluzole, providing only a moderate extension of patient life [10]. With the number of cases expected to rise to nearly 400,000 world-wide by 2040 [11], ALS represents a large unmet medical need for which many drugs are in development [12]. The use of validated and qualified biomarkers in ALS clinical trials could greatly increase the informative value of any conducted trial regarding whether the target was properly engaged (pharmacodynamic biomarker), the therapeutic potential of the drug or target (pharmacodynamic, prognostic biomarker), as well as assist in population stratification for likely responders to a specific treatment in this heterogeneous disease (predictive biomarker). Better biomarkers would assist in differentiating ALS from other diseases with similar presentation and the earlier diagnosis of ALS will allow patients to receive treatment sooner, as well as be eligible for clinical trials sooner (diagnostic biomarker). Furthermore, a biomarker that tracks with disease would aid in disease classification, prognostication, and assist in the perhaps personalized need for clinical intervention, as ALS phenotypes are largely heterogeneous [13].

Characteristics of an ideal biomarker for ALS include a high correlation with a disease process in the central nervous system (CNS) or with disease state / progression, stable throughout the day, high sensitivity and specificity depending on its use, and ease of obtaining samples, (e.g. blood) [14]. Biomarker research in ALS has led to the identification of p75^ECD^ from urine [15], phosphorylated neurofilament heavy (pNfH) in cerebrospinal fluid (CSF) [16], and neurofilament light (NfL) in serum [17] as diagnostic, prognostic, and pharmacodynamic biomarkers. However, these three are not unique to ALS, while potentially having a role in prognostication and disease monitoring, these biomarkers have low specificity for ALS. These biomarkers are also dependent on axonal loss and may reflect later stages of degeneration and therefore miss the spectrum of disease activity. Finally, CSF extraction for pNfH, while useful for diagnosis, does involve what can be a difficult procedure for some patients and serial lumbar puncture is unrealistic outside of clinical research. To develop an ideal biomarker for ALS a more rounded test or panel of tests is needed that is easy to reproduce, addresses the spectrum of disease, and is simple to obtain multiple specimens. To address the spectrum of disease activity this biomarker or group of biomarkers should focus on neuronal loss as well as other disease mechanisms.

Inflammation occurs in the CNS during ALS, and is also detected peripherally in blood as altered immune cell population abundance and released factors [18–20]. The potential causative or disease driving role of this inflammation in ALS is not fully clear, as there are studies that support both a primary or secondary role during ALS disease progression. Inflammatory biomarkers could reflect that bridge to other disease activity and, in combination with assays of neuronal degeneration, provide a better sense of disease subtypes and disease activity. In this review, we discuss the state of biomarkers of inflammation in ALS, focusing on peripherally detectable and cellular responses from blood cells, and provide new (combinatorial) directions for exploration that are now feasible due to technological advancements. We start with potential diagnostic biomarkers of inflammation in ALS, proceeding to prognostic biomarkers and conclude with how other biomarkers, pharmacodynamic and predictive, can assist in the design and interpretation of clinical trials in ALS.

### Diagnostic biomarkers

The diagnosis of ALS depends on the exclusion of other disease mimics first, and often takes over 12 months [21]. Hereditary neuropathy, acquired neuropathies, Myasthenia Gravis (MG), and central demyelinating disease can present similar to ALS and be difficult to fully exclude. This is compounded even more by the varied presentation of ALS (bulbar vs. limb onset, primary lateral sclerosis vs. progressive muscle atrophy, young onset vs. old onset), as well as by the addition of non-diagnostic characteristics such as flail-arm syndrome and frontotemporal involvement [13]. A robust and specific ALS diagnostic biomarker would aid to decrease the uncertainty and the time to diagnosis for all patients, allowing for earlier interventions and increasing the time window of eligibility to participate in clinical trials. With the exception of a few of these biomarkers (e.g. acetylcholine receptor antibodies in MG and MRI in multiple sclerosis) a specific and sensitive biomarker has been difficult to identify for these conditions, and even more so with (neuro) inflammation not solely occurring in ALS. As a result, therapeutic trials are based primarily on clinical scales and require longer observation to assess efficacy, thereby increasing cost and slowing drug development [22–25]. The initial hurdle for a diagnostic biomarker is to distinguish between ALS and controls (as the studies in Table 1 were designed to detect), but in practical use the biomarker(s) need to distinguish between ALS and other ALS-mimicking diseases, e.g. those that are excluded at diagnosis.

**Table 1:** Blood-based diagnostic biomarkers of inflammation in ALS

Measured biomarker(s)	Tissue and detection method	Mean disease duration at sample donation	Number of samples	Potential Diagnostic Value	Study Reference Clinical Trial ID
***Cell populations***					
Monocyte subpopulations	Monocyte isolation kit from peripheral blood, and flow cytometry	Not reported	*n* = 68 ALS (1st cohort) *n* = 55 controls (1st cohort) *n* = 100 ALS (2nd cohort) *n* = 60 controls (2nd cohort)	- CD14−/low/CD16+ monocytes decreased in ALS	Beers et al., 2020 [26]
Subpopulations of T cells, B cells, natural killer cells, and antigen presenting cells	Peripheral blood and FACS	2.48 years	*n* = 73 ALS *n* = 48 controls	- increased Th1 and Th17 cells in ALS - decreased Th2 and Treg cells in ALS - increased NK cells and monocytes in ALS	Jin et al., 2020 [27]
neutrophil-to-lymphocyte ratio (NLR)	From whole blood	Not reported	*n* = 80 ALS *n* = 80 matched controls(*n* = 41 ALS and n = 41 matched controls at follow-up 3-6 m after initial donation; *n* = 22 ALS patients and n = 22 matched controls at the third donation at least 3 months after the second)	- NLR was consistently elevated in ALS samples	Keizman et al., 2009 [28]
***Candidate-based soluble factor analysis***					
IL-2, IL-6, IL-10, IFN-γ, and TNF	Plasma, BioPlex	21.37 months (median)	n = 79 ALS *n* = 79 controls	- all measured cytokines were increased in ALS	Tortelli et al., 2020 [29]
CD14, LBP and CRP	Serum; ELISAs	Not reported	n = 68 ALS (1st cohort) *n* = 55 controls (1st cohort) *n* = 100 ALS (2nd cohort) n = 60 controls (2nd cohort)	- soluble CD14 increased in ALS (both cohorts) - LBP increased in ALS (both cohorts) - CRP increased in ALS (both cohorts)	Beers et al., 2020 [26]
IL-1b, IL-6, IL-10, IL-12, TNF,IFN-γ, IL17a, and IL-23	Serum; ELISAs	2.48 years	*n* = 73 ALS *n* = 48 controls	- increased IL-1b, IL-6, and IFN-γ in ALS - decreased IL-10 in ALS - no difference in TNF, IL-12, IL-17a, and IL-23	Jin et al., 2020 [27]
CD5L, Ficolin-3	Plasma; ELISAs	739.9 months (median)	*n* = 37 ALS *n* = 30 controls	CD5L and Ficolin-3 are increased in ALS	Mohanty et al., 2020 [30]
IL-2, IL-1b, TNF, IFN-γ and IL-4	Serum; ELISAs	40.4 months	*n* = 35 ALS n = 30 controls	- increased IL-4 and IL-1b in ALS - decreased IFN-γ in ALS - no difference in IL-2 and TNF	Polverino et al., 2020 [31]
MCP-1, eotaxin-1, IL-18, TNF, CRP, IL-1, sTREM2	Plasma; MSD assay	Not reported	*n* = 108 ALS n = 41 controls (*n* = 85 ALS with samples 2+ visits)	- MCP-1 and IL-18 are increased in ALS - sTREM2 is increased in ALS	Huang et a;., 2020 [32] NCT01495390
IL-6	Plasma; Chemokine assay	25.7 months	*n* = 82 ALS *n* = 43 controls	Not upregulated in ALS (trend)	Pronto-Laborinho et al., 2019 [33]
IP-10, MCP-1, MIG, RANTES, IL-2, IL-4, IL-6, IL-8, IL-10, IL-17a, TNF, IGF-g, sTNFR1, sTNFR2	Plasma; cytometric bead array and ELISA	3 years	n = 68 ALS *n* = 62 controls over the first time point. *n* = 24 ALS at the second time point (6–12 months later)	IL-6 + IL-8: upregulated in ALS IL-2 (low) and IL-6 (high) predict ALS diagnosis	Prado et al., 2018 [34]
Gene expression of 45 genes	Serum; individual RT-qPCRs	Within the first 2 months of diagnosis	n = 22 sALS *n* = 13 controls	- ITGB2, INPP5D, SELL, ICAM1, MMP9 and TIMP2 are upregulated in ALS - CCL5, CXC5R, IL10, TGFB2, IL10RA, IL-6, CD2 and TRBC1 are downregulated in ALS	Andres-Benito et al., 2017 [35]
Gene expression of 37 brain-enriched and inflammation-associated microRNAs	Plasma; individual RT-qPCRs	1.8 years	*n* = 50 ALS *n* = 50 FTD n = 50 AD n = 50 PD n = 50 controls	miR-206/miR-31 and miR-206/ miR-125b and miR-99/ miR-338-3p most effectively differentiate between ALS and control	Sheinerman et al., 2017 [36]
CC-16	Plasma; ELISA	27 months	*n* = 81 ALS n = 30 controls	Upregulated in ALS	Pronto-Laborinho et al., 2017 [37]
TNF, MCP-1, IL6, IL8, IL2, IFN-γ, IL1-beta, IL10, IL4, IL5, IL17, TNFR1, ELAM-1	Plasma; different per individual dataset (25 studies)	different per individual dataset (25 studies)	pooled *n* = 812 ALS pooled *n* = 639 controls	TNF, TNFR-1, IL-6, IL-1β, and IL-8 levels were elevated in ALS.	Hu et al., 2017
					[38]
TNF, IL-8, IL-6, IL-10	Serum; multiplex assay	Not reported	*n* = 19 ALS *n* = 10 controls	- IL-6 was increased in ALS. - IL-8 was increased in ALS.	Blasco et al., 2016 [39]
IL-1β, IL-18, IL-33, IL-37, IL-1Ra, sIL-1R2, IL-18BP, sIL-1R4	Serum; individual ELISAs	11.32 months	*n* = 144 sALS *n* = 40 controls	- IL-18 was increased in ALS. - IL-18BP was increased in ALS.	Italiani et al., 2014 [40]
IL-33, soluble ST2	Serum; individual ELISAs	Not reported	n = 42 ALS *n* = 38 controls	- IL-33 was increased in ALS - soluble ST2 was decreased in ALS	Lin et al., 2012 [41]
IL-17A	Serum; individual ELISA	23.4 months	*n* = 32 ALS *n* = 14 controls	IL-17A was increased in ALS.	Fiala et al., 2010 [42]
kynurenine pathway (tryptophan, picolinic acid)	Serum; HPLC, gas chromatography mass spectrometry	Not reported	*n* = 140 ALS n = 35 controls	- TRP and KYN is increased ALS - PIC is decreased in ALS	Chen et al., 2010 [43]
eotaxin, eotaxin-3, IL-8, IP-10, MCP-1, MCP-4, MDC, MIP-1b, TARC	Serum; solid-phase sandwich immuno-assay	Not reported	*n* = 20 ALS *n* = 20 controls (non-ALS neurologic)	No differences.	Kuhle et al., 2009 [44]
TNF, IFN-γ, and NO	Serum; individual ELISAs and NO by determining nitrite and nitrate levels	12 months	*n* = 22 ALS n = 20 controls	TNF, IFN-γ, and NO were all increased in ALS	Babu et al., 2008 [45]
RANTES	Serum; individual ELISA	Not reported	n = 20 ALS n = 14 NIND n = 13 controls	RANTES was increased in ALS serum	Rentzos et al., 2008 [46]
MCP-1	Serum; individual ELISA	19.4 months	*n* = 27 ALS n = 30 NIND	MCP-1 was increased in ALS	Baron et al., 2005 [47]
MCP-1	Serum; individual ELISA	8 months (median)	*n* = 29 ALS *n* = 11 controls	MCP-1 was not altered in ALS	Wilms et al., 2005 [48]
wide-range C-reactive protein (wrCRP)	From whole blood	Not reported	n = 80 ALS *n* = 80 matched controls	- wrCRP was consistently elevated in ALS samples	Keizman et al., 2009 [28]
**Unbiased analyses of serum or plasma**					
miRNA gene expression	Plasma; next generation sequencing on neural-enriched extracellular vesicles	Not reported	n = 10 + 10 (replication set) ALS *n* = 10 + 10 (replication set) controls	Eight miRNAs were differentially expressed between ALS samples and controls after replication. This included miR-146a-5p, which was upregulated in ALS samples and is associated with inflammation (monocytes) [49].	Banack et al.;. 2020 [50]Some samples from NCT03580616.
Protein abundance detectable by mass spectrometry	Plasma; mass spectrometry	747 months (median)	*n* = 42 ALS *n* = 18 controls	30 proteins are differentially detected between ALS and controls. IPA analysis identified two networks of interacting proteins that differ between ALS and controls; IL-1 and NFkB.	Xu et al., 2018 [51]
***Blood-derived cells,*** **in vitro** ***assays***					
Transcriptomic analysis	Gene expression of blood monocyte-derived macrophages, by RNAseq and RT-qPCR	Not reported	n = 5 controls n = 5 sALS n = 5 C9-ALS	- Increased type I interferon signature (pathway analysis) - increased gene expression of MX1, OASL, OAS2, IF44L	McCauley et al., 2020 [52]
Cell surface expression of VLA4, TLR4, CXCR3, CCR5, CXCR4, IFN-γ, CCR2, CD11B	Flow cytometry on blood-isolated T-cells, B-cells, monocytes, and NK cells	Not reported	n = 10 ALS n = 10 controls	- CXCR4, CXCR3, CCR2, CCR5 increased on ALS T-cells. - CD11B, CCR2 decreased on ALS monocytes - The combination of the analyzed markers could significantly predict the categorization into ALS or healthy donors, with CXCR3 and CCR5 on T cells comprising the strongest predictors.	Perner et al., 2018 [53]
Number of migrating cells	Boyden chamber. All cells migrated to the lower well after 2.5 h were stained using lineage antibody and counted by flow cytometry.	Not reported	n = 10 ALS n = 10 controls	More ALS CD45+ cells chemotaxis with IP-10 chemoattractant.	Perner et al., 2018 [53]
Frequency of myeloid dendritic cells	Flow cytometry (CD1c^high^CD19−)	Not calculated	n = 20 ALS n = 10 healthy donors	- Less circulating myeloid dendritic cells in ALS. - Increased CD62L expression on circulating myeloid dendritic cells in ALS.	Rusconi et al., 2017 [54]
Concentrations of TNF, IL-1β, IL-6, IL-12p40, IL-8, CCL2 and IL-10 in BDCA1+ DC supernatants	Circulating myeloid dendritic (CD1c^high^) cells stimulated with LPS.	Not calculated	*n* = 52 ALS *n* = 36 healthy donors *n* = 25 other neurological controls.	Higher levels of IL-8 and CCL-2 upon LPS- stimulation in ALS dendritic cells	Rusconi et al., 2017 [54]
116 leukocyte populations and phenotypes from lymphocytes, monocytes, and granulocytes	Peripheral blood immunophenotyping by flow cytometry	21.6 months	n = 80 ALS *n* = 50 controls	- 32 leukocyte phenotypes altered in ALS - elevated cell counts of granulocytes, NK cells and T cells in ALS - ALS patients were clustered into a profile distinct from controls primarily due to differences in multiple T cell phenotypes, CD3 CD56 T cells and HLA-DR on monocytes.	Gustafson et al., 2017 [55]
Transcriptomic analysis	RNA sequencing of blood monocytes	Not reported	n = 43 ALS n = 22 controls	ALS monocytes demonstrated a unique inflammation-related gene expression profile, the most prominent of which, including IL1B, IL8, FOSB, CXCL1, and CXCL2	Zhao et al., 2017 [56]
IL1-b, IL-6, IL-8, IL-10, GM-CSF, and TNF and TGF-b1, −b2, and -b3	By Luminex xMAP on supernatants from PBMCs or macrophages cultured overnight (non-stimulated, and SOD1-stimulated)	Not reported	1 discordant twin pair	- In non-stimulated conditions the supernatants from the ALS PBMCs increased IL-6, TNF, and IL-1.	Lam et al., 2016 [57]
Immune and cytokine profiling	freshly collected, un-stimulated cells by flow cytometry, on peripheral monocytes and T lymphocytes.	2.4 years	n = 24 ALS n = 25 controls	Th1-, Th17-, and IL-6-driven inflammation increased in ALS.	Saresella et al., 2013 [58]
90 inflammatory genes	qPCR analysis from isolated PBMCs	25.4 months	n = 10 ALS *n* = 4 controls	- 50% of the ALS patients had ‘strong inflammation’ (upregulation of IL-1, IL-6, IL23a, PTGS2, MMP1, CCL20, CXCL3, CXCL5 and CXCR4; downregulation of CXCL9, CXCL10, and CXCL11), the other 50% had ‘weak inflammation’. - all ALS patients had an ‘ALS signature’ with 4-fold increase of MMP1, CCL7, CCL13 and CCL24.	Fiala et al., 2013 [59] No NCT reported
IL-1b, IL-2, IL-4, IL-5, IL-6, IL-7, IL-8, IL-10, IL-12, IL-13, IFN-γ, GM-CSF, TNF	PBMC cultures, non-stimulated and stimulated with SOD1 protein, supernatants analyzed by R&D Systems High Sensitivity Human Inflammation Multiplex-Kit	26.9 months	n = 8 sALS n = 4 controls n = 1 unaffected twin of sALS patient	- 4 sALS patients had increased expression of TLR2 and CD14; ALOX5, PTGS2 and MMP1; IL1α, IL1β, IL6, IL36G, IL8 and TNF; CCL3, CCL20, CXCL2, CXCL3 and CXCL5. - 4 sALS patients had a decrease in the expression of PPARG, PPARA, RARG, HDAC4 and KAT2B; IL6R, IL6ST and ADAM17; TNFRSF11A; MGAT2 and MGAT3; PLCG1; CXCL3. - Difference identified between rapid ALS and slow ALS or controls. No diff between slow ALS and controls.	Mizwicki et al., 2012 [60]
monocyte and lymphocyte populations and activation	Surface expression, measured by flow cytometry from monocytes isolated from whole blood	4–93 months (range)	n = 38 sALS *n* = 28 controls	- increased percentage of CD4+ cells in ALS - increased mean CD14-HLD-DR expression in ALS - increased percentage of CD14 and CD16+ cells in ALS - increased serum IgG in ALS - decreased serum IgM in ALS	Zhang et al., 2005 [61]

*AD* Alzheimer’s Disease, *ALS* amyotrophic lateral sclerosis, *ALSFRS*-R ALS functional rating scale revised, *C9-ALS* ALS due to the harboring of the C9orf72 hexanucleotide repeat expansion, *CC-16* club cell protein 16, *CD14* cluster differentiation 14, *CD5L* cluster differentiation 5 ligand, *CMAP* compound muscle action potential, *CRP* c reactive protein, *DC* dendritic cells, *ELISA* enzyme-linked immunosorbent assay, *FACS* Fluorescence-activated cell sorting, *FTD* frontal temporal dementia, *FoxP3* Forkhead Box P3, *FVC* forced vital capacity, *HLA-DR* Human Leukocyte Antigen – DR isotype, *HPLC* High Performance Liquid Chromatography, *IFN-γ* interferon gamma, *IGF-g* Insulin-like growth factor gamma, *IgG* Immunoglobulin G, *IgM* Immunoglobulin M, *IL* interleukin, *IL-1RA* interleukin 1 receptor agonist, *IL-18BP* interleukin 18 binding protein, *IP-10* Interferon gamma-induced protein 10, *LBP* Lipopolysaccharide binding protein, *LPS* lipopolysaccharide, *MIP-1β* Macrophage inflammatory protein 1 beta, *MCP-1* Monocyte Chemoattractant Protein 1, *MIG* monokine induced by gamma interferon, *MMP* Matrix Metalloproteinases, *MSD* Meso Scale Discovery (multiplexing), *NFkB* nuclear factor kappa-light-chain-enhancer of activated B cells, *NIND* non-inflammatory neurological disorder, *NK* natural killer cells, *NLR* neutrophil-to-lymphocyte ratio, *NO* nitric oxide, *PBMC* peripheral blood mononuclear cell, *PD* Parkinson’s Disease, *RANTES* Regulated upon Activation, Normal T Cell Expressed and Presumably Secreted, *NA* ribonucleic acid,

*RT-qPCR* real time quantitative PCR, *sALS* sporadic amyotrophic lateral sclerosis, *SOD1* super oxide dismutase 1, *sTNFR* soluble TNF receptor, *sTREM2* soluble Triggering Receptor Expressed On Myeloid Cells 2, *TNF* tumor necrosis factor, *TNFR* tumor necrosis factor receptor, *Tregs* T regulatory cells, *wrCRP* wide-range c reactive protein

Inflammation as a biomarker in ALS, historically, was studied in context of the spinal cord disease. Inflammation-based biomarkers started with candidate-based analyses of cerebrospinal fluid (CSF), assessing cell populations such as lymphocytic infiltrates and secreted factors of the CNS [19]. The detection of peripheral inflammation in blood samples of ALS patients [20] led to the assessment of these blood-based markers of inflammation as potentially diagnostic biomarkers. Initially, studies were conducted on candidate-based cell populations, such as the increased neutrophil-to-lymphocyte ratio in ALS [28], which has potential as a diagnostic biomarker in multiple sclerosis [62], and soluble factors (Table 1). In-depth immunophenotyping by flow cytometry identified 116 distinct blood-derived cell populations of which 32 populations were altered, including elevated cell counts of granulocytes, NK cells and T cells in ALS samples compared to controls [55], and was confirmed by others [27]. Technological advancement led to the multiplexing of many soluble factors on the same sample simultaneously, but this also did not identify a robust ALS diagnostic biomarker (as of yet). Although candidate-based analyses suggest inflammatory pathways and factors (e.g. cytokines) may be differentially expressed in ALS, the variability among the reports of these measurements is high, with the most commonly measured cytokines are reported, in different studies, to be upregulated and/or downregulated and/or unaltered in ALS, including C-reactive protein (CRP) [26, 28, 32], interleukin 6 (IL-6) [27, 29, 33–35, 38–40, 56], IL-8 [32, 34, 38, 39, 44, 56], tumor necrosis factor (TNF) [27, 29, 31, 32, 34, 38, 39, 56], IL-1β [27, 31, 32, 38, 40], IL-17 [27, 32, 40], IL-33 [40, 41], IL-10 [27, 32, 34, 39], monocyte chemoattractant protein 1 (MCP-1) [34, 38, 44, 47, 48], and interferon gamma (IFNγ) [27, 31, 34, 45, 56] (Table 1). Please note that many of these studies are underpowered ‘pilot’ studies that require replication. To minimize inter-sample variability simultaneous combinations of single factors were assessed to strengthen the diagnostic value of the combined biomarkers, e.g. IL-2 and IL-6 [34], or microRNA (miRNA) pairs (ratios) [36]. (Please note, that these specific examples of combined markers results have not (yet) been replicated.)

With many of the subpopulations of blood cells producing different expression levels of genes or soluble factors, these analyses in blood provide an averaged data point, losing the cell-type specific resolution of the obtained data. This may partly explain why there is no established diagnostic biomarker for ALS (yet) by using single factor analyses, in addition to the other possible explanations including technology used [63], sample sizes, co-morbidities, (anti-inflammatory) medications, and disease state at biosample donation [64].

Assessing the gene or protein expression of specific cell populations (i.e. not using plasma, serum, or peripheral blood mononuclear cells (PBMCs) in a bulk analysis) provides higher resolution data, and has identified more detailed potential biomarkers. For instance, transcriptomic analyses of monocytes identified an unique inflammation-related gene expression profile, including altered IL-1B, IL-8, FOSB, C-X-C Motif Chemokine Ligand 1 (CXCL1), and CXCL2 gene expression, which distinguished ALS from control samples [56]. By differentiating blood monocytes into macrophages and conducting transcriptomic analyses on these cells McCauley et al., identified an impaired type I interferon response in the gene expression pathway analysis of ALS cells harboring the *C9orf72* repeat expansion [52]. Additionally, in-depth immunophenotyping of 80 ALS and 50 control samples by Gustafson et al., detected an ALS immune cell profile of 32 leukocyte populations, including T cell phenotypes, and the levels of the activation marker Human Leukocyte Antigen DR isotype’s (HLA-DR) expression on monocytes [55], which has been confirmed by others [61]. To further assess potential biomarkers in a cell-type specific manner, in vitro assays designed to detect the inflammatory-response post stimulation by lipopolysaccharide (LPS) identified elevated levels of IL-8 and CCL-2, but not of TNF, IL-1β, IL-6, IL-12p40, IL-8, C-C Motif Chemokine Ligand 2 (CCL2) and IL-10, in the conditioned media from ALS dendritic cells [54], a cell type closely related to monocytes. Interestingly, miRNA analysis of neural-enriched extracellular vesicles from plasma identified that miR-146a-5p, also associated to monocyte inflammatory response [49], is increased in ALS [50]. Alternative assays are used by stimulating cells with SOD1 proteins [57, 60] or staphylococcal enterotoxin B (SEB) [58] to increase the detectable levels of cytokines, often with a low number of samples included per study, and have not (yet) identified a biomarker.

Although beyond the scope of this review, the reoccurring indication of (activated) monocyte biology as a diagnostic biomarker in ALS begs the question how this cell type may contribute to ALS disease pathophysiology. One such direction may be the increased production of chitinases by monocyte-derived macrophages [65], and/or by the secretion of cytotoxic cytokines (reviewed in [18]). More research is needed to further our understanding of the contribution of monocyte biology to ALS.

Although some of these biomarkers, or biomarker profiles, distinguish ALS from controls, more research is needed to assess their usefulness to ALS, including their sensitivity, specificity, and robustness to assist in the diagnosis of ALS as many cytokines are also elevated or decreased in other indications. In view of the promising results from cell-type specific analyses, future work could include cell-type specific analyses of circulating or in vitro-invoked inflammatory responses.

### Prognostic biomarkers

In clinical trials, an FDA accepted endpoint of an effective drug for ALS is the slowing of disease-related decline, stabilization, improvement of function in daily activities as measured, for example, by the ALS Functional Rating Scale-Revised (ALSFRS-R) or a similar scale [66]. With the progressively increased inflammation and the progressive decline in ALSFRS-R during disease progression, blood-based markers of inflammation may be exceptionally suitable as prognostic biomarkers. These would predict the rate of decline in a patient, and potentially distinguish between disease sub groups (slow and rapid progressors) and adjust clinical trial eligibility criteria to minimize inter-subject variability. Disease duration is often defined as the number of months post disease onset or diagnosis and survival [30, 67]. As there is significant heterogeneity in the rate of ALS disease progression [68], ‘disease duration’ may not reflect changes in motor function or independence in daily living as well as the ALSFRS-R, or other quantifiable scales or measurements.

During disease progression, electromyographical measures are altered, including a reduction in the compound motor action potential (CMAP) of the phrenic nerve, as well as a reduction in respiratory measures such as Forced Vital Capacity (FVC). These measurements were both not correlated to CC-16 expression [37], but had a weak inverse correlation with plasma IL-6 expression [33]. The broad and responsive inflammation marker CRP is reported to have a weak correlation with ALS disease duration [26, 28, 67, 69], as well as CD5L, TNF, IFNγ, and nitric oxide (NO) [30, 45]. Strikingly, studies assessing pro- and/or anti-inflammatory cytokines (IP-10, MCP-1, MIG, RANTES, CC-16 IL-2, IL-4, IL-6, IL-8, IL-10, IL-17a, TNF, IFNγ, sTNFR1, sTNFR2, IL-1β, IL-18, IL-33, IL-37, IL-1Ra, sIL-1R2, IL-18BP, sIL-1R4) in the blood of ALS patients (Table 2) were unable to detect a correlation with ALSFRS-R scores of patients that they assessed at the time of biosample donation [27, 33, 34, 37, 40, 70]. In addition, Devos et al. assessed the levels of IL-6 in the plasma of 109 ALS patients at the beginning of a clinical trial (NCT00868166) with ALSFRS-R measurements up to 18 months. Although the authors identified a predictive correlation with non-inflammation blood-based markers with ALSFRS-R rate of decline (NfL, ferritin, 4-HNE, 8-OHdG), IL-6 levels at baseline were not predictive [70]. Interestingly, a recent study with 79 ALS patient samples, detected a correlation between IL-6 and the ALSFRS-R [29]. These studies indicate that a bulk measurement of a cytokine in plasma or serum may not provide sufficient resolution as a prognostic biomarker, similar to discussed for diagnostic biomarkers. In support of this idea, increasing the resolution of measurement by assessing the expression levels of cytokines, perhaps in combination, on specific blood-based immune cell populations provides potential. As an example of this approach, during the clinical trial assessing the safety of NP001 (regulator of macrophage and monocyte activation) in ALS (NCT01091142), the expression levels of the monocyte activators CD16 and HLA-DR on the surface of monocytes were measured throughout the clinical trial. Although these measurements were not correlated with the score of the ALSFRS-R on the day of biosample donation, they were correlated with ALSFRS-R rate of decline. Moreover, the combination of both markers simultaneously correlated with the ALSFRS-R rate of decline more strongly [72]; a higher the level of activation correlated with a more rapid ALS disease progression [61, 72].

**Table 2:** Blood-based prognostic biomarkers of inflammation in ALS

Measured biomarker	Method of detection	Mean disease duration at sample donation	Number of samples	Potential Prognostic Value	Study Reference Clinical Trial ID
**Blood-derived markers**					
IL-2, IL-6, IL-10, IFN-γ, and TNF	Plasma, BioPlex	21.37 months (median)	*n* = 79 ALS	IL-6 correlated with ALS-FRS-R and Manual Muscle Testing.	Tortelli et al., 2020 [29]
CD14, LBP and CRP	Serum; ELISAs	Not reported	n = 68 ALS (1st cohort) *n* = 100 ALS (2nd cohort)	- soluble CD14 correlated to burden of disease and progression rate (both cohorts) - LBP correlated to burden of disease and progression rate (2nd cohort) - CRP correlated to burden of disease and progression rate (2nd cohort)	Beers et al., 2020 [26]
IL-1b, IL-6, IL-10, IL-12, TNF, IFNg, IL17a, and IL-23	Serum; ELISAs	2.48 years	n = 73 ALS	- IL-1b was increased in fast progressive ALS - IL-6 correlated with disease duration (weak correlation) - IL-1b correlated with the ALSFRS-R slope	Jin et al., 2020 [27]
CD5L, Ficolin-3	Plasma; ELISAs	739.9 months (median)	*n* = 37 ALS	- CD5L was correlated with disease duration and survival (not with ALS-FRS). - Ficolin-3 was not correlated to disease parameters.	Mohanty et al., 2020 [30]
CRP	Serum; standard laboratory tests	Retrospective study of newly diagnosed ALS patients with up to 5 years of follow-up (average 2.36 years).	*n* = 399 ALS(*n* = 122 very fast progressors, *n* = 88, medium progression, *n* = 189 slow progression)	- Patients with a higher CRP (log-transformed) at baseline had a higher risk of mortality. - Patients with a higher CRP (log-transformed) than at baseline had a higher risk of mortality. - CRP (log-transformed) increases in the last few months prior to death in the medium and fast progressing patients)	Sun et al. 2020 [67]
CRP	Serum; standard laboratory tests		*n* = 384 ALS *n* = 116 ALS (replication study)	Increased serum CRP is correlated with an increased rate of functional decline.	Lunetta et al., 2017 [69] (NCT01281631)
wide-range C-reactive protein (wrCRP) concentrations	From whole blood	Not reported	n = 80 ALS (*n* = 41 ALS at follow-up 3-6 m after initial donation; *n* = 22 ALS patients at the third donation, and at least 3 months after the second)	- Correlation between the ALSFRS-R and the wrCRP concentration at the first examination.	Keizman et al., 2008 [28]
IL-6	Plasma; Human Magnetic Luminex Screening Assay	Collection at 1, 6, 12, and 18 months into the clinical trial	*n* = 109 ALS participants measured over 4 time points	No correlation with ALS-FRS-R	Devos et al., 2019 [70] NCT:00868166
IL-6	Plasma; Bio-Plex Pro Human Chemokine assay.	25.7 months	n = 82 ALS	Reduction in phrenic nerve CMAP amplitude and FVC was correlated with increased IL-6 levels	Pronto-Laborinho et al., 2019 [33]
IP-10, MCP-1, MIG, RANTES, IL-2, IL-4, IL-6, IL-8, IL-10, IL-17a, TNF, IGF-g, sTNFR1, sTNFR2	Plasma; cytometric bead array and ELISA	3 years	n = 68 ALS *n* = 24 ALS at the second time point (6–12 months later)	No correlation with ALS-FRS-R for any tested markers.	Prado et al., 2018 [34]
CC-16	Plasma; ELISA	27 months	n = 81 ALS	No correlation with age, onset region, disease duration, functional status, FVC, and PhrenAmpl.	Pronto-Laborinho et al., 2017 [37]
IL-1β, IL-18, IL-33, IL-37, IL-1Ra, sIL-1R2, IL-18BP, sIL-1R4	Serum; individual ELISAs	11.32 months	n = 144 sporadic ALS	No correlations with the ALS-FRS-R were detected.	Italiani et al., 2014 [40]
TGF-b, IL-6, TNF, IL-17A	Serum; individual ELISAs	From 3 to 96 months	*n* = 21 ALS	No correlation between the 4 cytokines and months after diagnosis.	Liu et al., 2012 [71]
TNF, IFN-γ, and NO	Serum; individual ELISAs and NO by determining nitrite and nitrate levels	12 months	n = 22 ALS	Correlation between TNF-a, IFN-γ, and NO levels and disease duration	Babu et al., 2008 [45]
RANTES	Serum; individual ELISA	Not reported	n = 20 ALS	No correlation with serum RANTES and disease duration.	Rentzos et al., 2008 [46]
TGF-b, IL-6, TNF, IL-17A	Serum; individual ELISAs	From 3 to 96 months (range)	n = 21 ALS	TGF-β and IL-6 were increased in some patients since the onset of symptoms, whereas IL-17A and TNF-α levels were increased only in the mid-course of the disease (no statistics reported)	Liu et al., 2012 [71]
**Blood-derived cells, in vitro assays**					
Monocyte subpopulations	Monocyte isolation kit from peripheral blood, and flow cytometry	Not reported	n = 68 ALS (1st cohort) *n* = 100 ALS (2nd cohort)	- CD14−/low/CD16+ monocytes negatively correlated with disease burden and rate of progression in ALS	Beers et al., 2020 [26]
Subpopulations of T cells, B cells, natural killer cells, and antigen presenting cells	Peripheral blood and FACS	2.48 years	n = 73 ALS	- increased NK cells in slow vs fast progressive ALS - no difference between slow and fast progressive ALS for any other cell population - Th1/Th2 ratio correlated with the ALSFRS-R slope - Th17/Treg ratio correlated with the ALSFRS-R	Jin et al., 2020 [27]
Concentrations of TNF, IL-1β, IL-6, IL-12p40, IL-8, CCL2 and IL-10 in DC supernatants	Circulating myeloid dendritic (CD1c^high^) cells stimulated with LPS.	Not calculated	n = 52 ALS	Inverse correlation between the time from onset to diagnosis and the levels of IL-6 secretion induced by LPS.	Rusconi et al., 2017 [54]
116 leukocyte populations and phenotypes from lymphocytes, monocytes, and granulocytes	Peripheral blood immunophenotyping by flow cytometry	21.6 months	n = 80 ALS	Different immuno-phenotypic markers associate with clinical parameters, incl. Survival, in the 2 ALS immune profiles.	Gustafson et al., 2017 [55]
Transcriptomic analysis	RNA sequencing of blood monocytes	Not reported	n = 43 ALS	ALS monocytes from rapidly progressing patients had more proinflammatory DEGs than monocytes from slowly progressing patients.	Zhao et al., 2017 [56]
Transcriptomic and methylation analysis	RNAseq and RRBS on PBMCs	Not reported	1 discordant twin pair	- Higher abundance of CD14 macrophages in ALS over time - Lower abundance of T cells in ALS over time	Lam et al., 201 6[57]
CD16 and HLA-DR	Surface expression, measured by flow cytometry from monocytes isolated from whole blood	24.1 months	n = 24 ALS	- CD14 correlated with ALS-FRS-R rate of change - CD14/HLA-DR correlated with ALS-FRS-R rate of change	Miller et al., 2014 [72] NCT01091142, Ph1 NP001 in ALS
monocyte and lymphocyte populations and activation	Surface expression, measured by flow cytometry from monocytes isolated from whole blood	4–93 months (range)	n = 38 sALS	- HLA-DR expression on CD14+ cells correlated with ALSFRS-R	Zhang et al., 2005 [61]
Leukocyte number and expression of FoxP3, TGF-b, IL-4, Gata-3, IL-10, Tbx21, IFN-γ	T-lymphocytes assessed by flow cytometry, and gene expression by RT-qPCR	Not reported	*n* = 54 ALS *n* = 102 ALS (replication)	- Number of Tregs and their FoxP3 protein expressions were reduced in rapidly progressing ALS patients and inversely correlated with progression rates (AALS). - The mRNA levels of FoxP3, TGF-b, IL4 and Gata3, were reduced in rapidly progressing patients and inversely correlated with progression rates. - FoxP3 and Gata3 were indicators of progression rates. - No differences in IL10, Tbx21, or IFN-γ expression were found between slow and rapidly progressing patients.	Henkel et al., 2012 [73]

*ALS* amyotrophic lateral sclerosis, *ALSFRS-R* ALS functional rating scale revised, *CC-16* club cell protein 16, *CCL2* C-C Motif Chemokine Ligand 2, *CD5L* cluster differentiation 5 ligand, *CD14* cluster differentiation 14, *CMAP* compound muscle action potential, *CRP* c reactive protein, *DC* dendritic cells, *ELISA* enzyme-linked immunosorbent assay, *FACS* Fluorescence-activated cell sorting, *FVC* forced vital capacity, *FoxP3* Forkhead Box P3, HLA-*DR* Human Leukocyte Antigen – DR isotype, *IFN-γ* interferon gamma, *IGF-g* Insulin-like growth factor gamma, *IL* interleukin, *IL-18BP* interleukin 18 binding protein, *IL-1RA* interleukin 1 receptor agonist, *IP-10* Interferon gamma-induced protein 10, *LBP* Lipopolysaccharide binding protein, *LPS* lipopolysaccharide, *MIG* monokine induced by gamma interferon, *MCP-1* Monocyte Chemoattractant Protein 1, *NK* natural killer cells, *NO* nitric oxide, *PBMC* peripheral blood mononuclear cell, *RANTES* Regulated upon Activation, Normal T Cell Expressed and Presumably Secreted, *RNA* ribonucleic acid, *RRBS* Reduced representation bisulfite sequencing, *RT-qPCR* real time quantitative PCR, *sIL-1R* soluble IL receptor, *sTNFR* soluble TNF receptor, *Tbx21* T-Box Transcription Factor 21, *TGF-β1* tumor growth factor beta 1, *TNF* tumor necrosis factor, *Tregs* T regulatory cells, *wrCRP* wide-range c reactive protein

In addition, in-depth immunophenotyping of 116 leukocyte populations from blood-derived immune cells identified a monocyte immune profile, as well as an T-reg immune profile, in a subpopulation of ALS patients correlated to the ALSFRS-R and survival [55]. This subpopulation accounts for ~ 39% of the tested cohort, were generally younger (54.8 vs. 60.5 years old), were more likely familial ALS (26.1% vs 2.8%), and survived longer than patients without this specific immune profile (344 vs. 184 weeks) [55], indicating that this specific inflammatory profile may be protective. Moreover, specifically assessing the transcriptomic profile of blood-derived monocytes additionally also identified alternative gene expression in monocytes between patients with a rapid and slow disease progression [56].

The value of cell population specific studies is further illustrated by the study from Henkel et al., in which they define a slow and rapid disease progression on basis on the Appel ALS score (AALS) [74]. The abundance of T-regs and the their FoxP3 protein expression was lower in rapidly progressing ALS patients, and was inversely correlated with disease progression rates determined by the AALS [73]. In addition, the gene expression levels of FoxP3, TGF-b, IL-4 and Gata3 from leukocytes were also decreased in rapidly progressing patients, and also inversely correlated to progression rate [73], whereas when measured in plasma the levels of TGF-b [71] and IL-4 [34] were not correlated to disease progression.

The subpopulations described by Jin et al., Beers et al., Henkel et al., Zhao et al., and Gustafson et al., imply that there are discernable subtypes of progression in ALS that are associated with inflammatory responses detectable in blood. Monitoring these responses could be used in population stratification for clinical trials, or as biomarkers that predict responders. The population-specific characteristics between these studies overlap in the identification of monocytes, T-regs and their responses as possible distinguishers of these subpopulations [26, 27, 55, 56, 73].

The future of prognostic biomarkers in ALS may include their use to predict disease progression rate, and thus clinical trial eligibility. Strides have been made to use a disease progression predicting algorithm to minimize variation and expand potential clinical trial eligibility [75, 76], as well as to perform post-hoc analyses to assess drug efficacy per participant, although these have not yet incorporated biological biomarkers. Future studies may determine whether inclusion of blood-based biomarkers (of inflammation) may increase the accuracy of the algorithm, e.g. for each of the subpopulations identified by immune profiles by Gustafson et al. [55]. In addition, with novel research, prognostic biomarkers may be applied to predict not only the disease progression after disease onset, but perhaps also predict phenoconversion from a pre-symptomatic to a symptomatic disease stage in high-risk individuals (e.g. those harboring a known ALS mutation) [77, 78]. A longitudinal study with healthy aging, mild cognitive impairment, and Alzheimer’s Disease participants over 5 years identified approximate time windows prior to phenoconversion and to the next disease symptom on the basis of biomarker changes detected many years prior [79]. In individuals at risk for ALS, NfL is increased in plasma and CSF ~ 12 months prior to symptom onset [80]. The future identification of additional prognostic biomarkers for phenoconversion in ALS may allow for an understanding of possible prevention intervention windows [77].

### Pharmacodynamic biomarkers

Despite the prediction that neuroinflammation contributes significantly to ALS pathophysiology [19], anti-inflammatory therapeutic strategies have failed in clinical trials in ALS [1]. The inclusion of readouts to confirm target engagement (pharmacodynamic biomarkers) in clinical trial design assist in confirming whether the therapeutic exerted the expected effect per participant throughout the treatment paradigm informing also target validity, and is highly recommended [81, 82]. In fact, the hypothetic inclusion of pharmacodynamic biomarkers in previous clinical trials may have aided in the understanding of the drugs or targets in ALS, and perhaps would have minimized clinical trials on the same target due to this increased knowledge. Although many therapeutics aim to target inflammation of the CNS, peripheral readouts may be established as a proxy readout. An example is monocyte activation as measurement of NP001 target engagement [72], or cytokine profiling in CSF and blood [83, 84]. Interestingly, a Phase 2 trial of tocilizumab (a humanized monoclonal antibody against the IL-6 receptor) in ALS (NCT02469896) assessed both mRNA profiling of PBMCs and CRP expression in blood and CSF, and although the transcriptomic readout was inadequate as a pharmacodynamic (or predictive) biomarker [84], the CRP levels were significantly altered by the treatment and may function as a pharmacodynamic biomarker.

Table 3 includes the biomarkers used in Phase 3 clinical trials targeting inflammation in ALS, as published or listed on clinicaltrials.gov supplemented with any peer-reviewed published information. Even in the case of targeting (neuro) inflammation with a medical product in ALS, few clinical trial designs include pharmacodynamic biomarkers (Table 3), although these may have been included at an earlier clinical stage. Perhaps due to the labor-intensive nature of in vitro assays, and the needed standardization or coordination between clinical sites, these paradigms have not (yet) been utilized as pharmacodynamic biomarkers in ALS during treatment (Table 3).

**Table 3:** Phase 3 clinical trials in ALS with a medical product targeting (neuro)inflammation

Intervention, Sponsor or Collaborators, Phase	Target or Mechanism of Action and Protocol	Outcome Measures	Pharmacodynamic Biomarker	Study results	Reference Clinical Trial ID
Drug: minocyclineSponsor: National Institute of Neurological Disorders and Stroke (NINDS) Phase: 3	- anti-inflammatory - daily dose for 9 months	- change in function as detected by the ALSFRS-R - changes in manual muscle testing (MMT), forced vital capacity (FVC, percent predicted), quality of life (QOL) and survival	none listed or described	- ALSFRS-R score deterioration was faster - (non-significant tendencies towards faster decline in FVC and MMT score, and greater mortality during the 9-month treatment phase - Quality-of-life scores did not differ between the treatment groups. - Non-serious gastrointestinal and neurological adverse events were more common in the minocycline group than in the placebo group, but these events were not significantly related to the decline in ALSFRS-R score.	Gordon et al., 2007 [3]NCT00047723
Drug: Granulocyte Colony Stimulating FactorSponsor: Tehran University of Medical Sciences Phase: 2/3	- G-CSF administered per subcutaneous injection - 5 days treatment with 3 month follow-up	- patient’s function - mobilizing bone marrow stem cells- amplitude of compound muscle action potential in ulnar and peroneal nerve- quality of life- muscle power	mobilizing bone marrow stem cells: - cluster of differentiation 34 (CD34) - white blood cell (WBC) counting	- no significant effect	Amirzagar et al., 2015 [4] NCT01825551
Drug: MediCabilis CBD Oil Sponsor: Gold Coast Hospital and Health Service, BOD Australia Phase: 3	- anti-inflammatory - treatment for 6 months	- difference in mean ALSFRS-R - difference in mean Forced Vital Capacity (FVC) - nature and number of adverse events - difference in mean Numeric Rating Scale for spasticity - difference in mean Numeric Rating Scale for pain total score - difference in mean Percentage of Total Weight Loss score - difference in mean ALS Specific Quality of Life- Revised	none listed	- not available yet (recruiting)	Urbi et al., 2019 [5] NCT03690791
Drug: Masitinib (4.5/3.0)Sponsor: AB Science Phase: 2/3	- microglia & mast cells through c-kit - 48 weeks	- change in ALSFRS-R - change of Forced Vital Capacity (FVC) - progression Free Survival - overall Survival	none listed or described	- “Normal progressor” subpopulation received a benefit from the drug on the ΔALSFRS-R and on the ALSAQ-40, FVC, and time-to-event analysis. - No differences were detected in the full sample (“Normal and Fast Progressor”).	Mora et al., 2020 [6] NCT02588677
Drug: Masitinib (6.0/4.5) Sponsor: AB Science Phase: 3	- microglia & mast cells through c-kit - 48 weeks	- ALSFRS-R - ALSAQ-40 - progression free survival - FVC - HHD	none listed	- not available yet (recruiting)	NCT03127267
Drug: ZilucoplanSponsor: Ra Pharmaceuticals Phase: 2/3	- complement C5 inhibitor - 24 weeks	- disease progression - respiratory function - muscle strength - survival	none listed	- not available yet (recruiting)	NCT04436497
Drug: VerdiperstatSponsor: Biohaven Pharmaceuticals, Inc. Phase: 2/3	- myeloperoxidase (MPO) enzyme inhibitor - 24 weeks	- disease progression - respiratory function - muscle strength - survival	none listed	- not available yet (recruiting)	NCT04436510
Drug: MN-166 (Ibudilast)Sponsor: MediciNova Phase: 2/3	- phosphodiesterase inhibitor (PDE4) - 52 weeks of treatment	- change from baseline in ALSFRS-R score at Month 12 - survival time - mean change of muscle strength measured by hand-held dynamometry - mean change from baseline on quality of life by ALSAQ-5 - mean change from baseline of functional activity by ALSFRS-R - responders, measured in percent of subjects overall, whose ALSFRS-R total score was stable or improved - time to survival - number of Participants with Treatment-Related Adverse Events - changes from Baseline in Laboratory Values	not specified (laboratory values)	- not available yet (recruiting)	NCT04057898
Biological: LenzumestrocelSponsor: Corestem, Inc. Phase: 3	- intrathecal autologous bone marrow-derived mesenchymal stem cells injections to minimize pro-inflammatory cytokines - Study drug injections twice in a 26-day interval followed by repeated three times study drug injections every three months.	- joint rank scores (CAFS, Combined Assessment of Functional and Survival) - ALSFRS-R score - time to event - Slow Vital Capacity (SVC)	Exploratory investigation of biological markers in plasma, blood and CSF. Comparison of change before and after treatment. Measurement cytokines: TGF-β1, IL-10, IL-6, TNF, MCP-1, IL-8, IL-1RA, MIP-1β, RANTES and IP-10 etc.	- not available yet (recruiting)	NCT04745299
Biological: RavulizumabSponsor: Alexion Pharmaceuticals Phase: 3	- complement inhibitor - 50 weeks	- change From Baseline ALSFRS-R Total Score - time To Ventilator Assistance-free Survival - change From Baseline In Slow Vital Capacity - incidence Of Treatment-emergent Adverse Events (TEAEs), Treatment-emergent Serious Adverse Events, And TEAEs Leading To Study Drug Discontinuation - change From Baseline In Muscle Strength As Assessed By Handheld Dynamometry - change From Baseline In Serum Neurofilament Light Chain	none listed	- not available yet (recruiting)	NCT04248465

*ALSAQ-5* five item ALS assessment questionnaire, *ALSAQ-4*0 forty item ALS assessment questionnaire, *ALSFRS-R* ALS functional rating scale revised, *CAFS* Combined Assessment of Functional and Survival, *CD34* cluster of differentiation 34, *c-kit* tyrosine-protein kinase Kit, *CSF* cerebral spinal fluid, *FVC* forced vital capacity, *HHD* hand-held dynamometry, *IL* interleukin, *IL-1RA* interleukin 1 receptor agonist, *IP-10* Interferon gamma-induced protein 10, *MCP-1* Monocyte Chemoattractant Protein 1, *MIP-1β* Macrophage inflammatory protein 1 beta, *MMT* manual muscle testing, *MPO* myeloperoxidase, *NINDS* National Institute of Neurological Disorders and Stroke, *PDE4* phosphodiesterase inhibitor 4, *QOL* quality of life, *RANTES* Regulated upon Activation, Normal T Cell Expressed and Presumably Secreted, *SVC* Slow Vital Capacity, *TEAEs* Treatment-emergent Adverse Events, *TGF-β1* tumor growth factor beta 1, *TNF* tumor necrosis factor, *WBC* white blood cell

### Predictive biomarkers

The strong heterogeneity of the ALS clinical phenotypes [85] and the identification of specific immune phenotypes and inflammatory responses that correlate with rate of disease progression [26, 55, 73], imply that subsets of ALS patients exist and that some may be particularly responsive to a therapeutic target and drug. A priori predictions of the potential responsiveness per patient to a therapeutic strategy could lead to clinical trial inclusion criteria on basis of such a predictive biomarker, and thus an enrichment of potentially responsive participants per clinical trial, as well as support personalized medicine endeavors [82]. For instance in ALS, the Phase 2 clinical trial for NP001, a monocyte and macrophage regulator, identified CRP levels as a predictor of responsiveness [69].

### Safety biomarkers

With the testing of novel medical products there is a need to identify potential safety biomarkers that will predict adverse events prior to the event occurring. An example is the monitoring of liver enzymes during the treatment with a drug that may cause liver damage, or particularly in those participants with a specific genetic polymorphism that affects drug metabolism to predict whether e.g. irreversible liver damage will occur. With the advancement of novel technologies in medical products, and with the genetic component of ALS, new technologies are developed with new (unknown) risk profiles, in particular biologics [86], including monoclonal antibodies, antisense oligonucleotides (ASOs), AAV-delivered gene therapies, and cell therapies. The safety risks can include excessive inflammation, immunogenicity, cytokine storms, and ultimately sepsis. Monoclonal antibody treatment, e.g. to address (neuro) inflammation in ALS, can give rise to cytokine release syndrome, which is an exaggerated systemic immune response involving the potential release of more than 150 inflammatory mediators [87]. This syndrome can be identified by monitoring cytokine levels in the blood post treatment, and can be treated promptly when detected [87]. ASO treatment has found increased application in clinical development in ALS; initially by targeting of the *SOD1* gene for ALS patients harboring an ALS-causing mutation in this gene in clinical trials [88], and with the additional development of ASO treatment for C9orf72 repeat expansion carriers (NCT03626012, NCT04288856) as well as the targeting of the CAG repeat expansion of the genetic disease modifier *ATXN2* (NCT04494256). Please note that these potential ASO treatments are still in clinical development and have not (yet) received marketing approval. These potential treatments involve the intrathecal injection of targeting ASOs, and provide the risk of infection [89], including meningitis, and of the above-described cytokine release syndrome, which has also been observed with ASO treatment [90]. Both of these safety risks can be detected early in blood for subsequent treatment. An alternative delivery of gene-based therapies is the use of viral vectors, e.g. adeno-associated virus (AAV), in preclinical development and as marketed medical products (e.g. Zolgensma for spinal muscular atrophy). Immunogenicity to the viral vector provides a risk to the efficacy of the potential therapy as well as a risk to adverse effects and complications from the treatment. For CNS bioavailable gene therapies this can include the neuroinflammatory response [91]. Treatment of ALS with cells that are intended to replace non-neuronal cells and/or support motor neuron health by controlling the milieu with trophic or anti-inflammatory factors have not (yet) demonstrated efficacy for ALS patients, but are in clinical development [92, 93]. These cell therapies may also harbor severe safety risks including immunogenicity and cytokine storms, which must be monitored and may be predicted with biomarkers. To this end, the use of blood-based inflammation biomarkers may be particularly useful to predict, and thereby potentially prevent, (serious) adverse events in clinical trials and/or in the initial or prolonged use of a marketed product.

## Conclusions

The peripheral inflammatory signature in ALS includes changes in cell population abundance, gene expression changes per cell population, and soluble factors such as cytokines; which elements reach pathologic significance is not at all clear. Therefore, it is not surprising that a single inflammatory marker derived from serum or plasma may not provide the needed sensitivity to distinguish between an ALS disease state, predict ALS disease progression, or assess whether a tested therapy is efficacious. The first potential solution is the inclusion of multiple readouts per sample to determine a combinatorial biomarker’s validity, which can be gene expression from the same cell [72], as well as combinations of different assays, including from cellular-based assays. In particular, the monocytic and T-reg pathway may be enriched for useful biomarkers in ALS. Conversely, monocytic and macrophage-based inflammation has been targeted therapeutically by NP001, and was not efficacious on the needed primary endpoints for FDA market approval in ALS [69, 72, 94]. Despite that targeting these pathways was not efficacious, these pathways may be enriched for useful biomarkers for future cell-specific and combined assessments, and may indicate disease processes that are more upstream than what was targeted. In particular, an in-depth assessment of monocyte-populations and their cell-specific transcription profiles (as described below), either by single cell sequencing or nuclear sequencing after monocyte-enrichment, may be a fruitful strategy for high-resolution biomarker detection. In addition, incorporating this combination approach with patient level clinical phenotype data could identify inflammatory traits and subgroups more responsive to selected therapies [13]. For example, the pattern of inflammation in upper motor neuron predominant disease may differ significantly from patients with lower motor neuron specific disease. This identification of novel associations may help to better classify disease and treatment approaches, allowing, ultimately, for stratification by inflammatory, or some other, biomarker trait.

The advanced technologies over the last decade allow for more detailed, in-depth, and higher resolution analyses, promoting the potential of combinatorial readouts from niche cell populations with e.g. the advances in single cell transcriptomic analyses which provides a large number of data points for all cell types in a sample, also for cells in blood and CSF in disease [95]. Current limitations of single cell sequencing involve the detection of poly-A adenylated gene expression transcripts only, ensuring that for assessing miRNAs or circRNAs per cell population, prior cell-sorting will be needed. miRNA studies on ALS serum, without cell-type specific sorting, also report variable results [96–98], reminiscent of the variation described above regarding cytokine analyses on serum. Cell type specific miRNAs and/or circRNAs may provide novel avenues for biomarker development in (neuro) inflammation [99] and in ALS [100]. These readouts can additionally be assessed in cell type specific in vitro assays, and both unstimulated and stimulated responses should be assessed, as the level of T-reg and monocyte activation is important [56, 61, 72].

In view of the studies discussed here, a compelling direction is the combination of single cell transcriptomic analyses on whole blood samples, in addition to cellular-based assays of a specific cell population. In addition, the combined biomarkers may gain in sensitivity and specificity when combined with other biomarkers, such as the established p75^ECD^ and/or neurofilament light. Other biomarkers under development include miRNAs, circRNAs, and the content of extracellular vesicles and exosomes from circulating blood cells or from in vitro assays, or other biomarkers such as neuroimaging (e.g. DTI or PET) and/or electrophysiological measurements (e.g. MUNIX) [64].

For the development of diagnostic and prognostic biomarkers in ALS of blood-based circulating factors (most cellular-based assays require fresh samples, as cell quality may be affected by long-term storage conditions [101]), many previously collected samples are available at biobanks and biorepositories. New sample collections e.g. during clinical trials of a potential therapeutic agent are recommended for monitoring, prognostic, pharmacodynamic, and predictive biomarkers, or those requiring fresh samples for cellular-based assays (as conducted in Table 2). During the collection of new samples, also across multiple clinical trial sites, it is essential that the samples are collected and prepared as similarly as possible, especially when intended to assess immune or inflammatory biomarkers. The variability during sample collection and preparation can result in variable levels of immune/inflammatory cell “activation”, which can introduce experimental artifacts into both the results and interpretations [102]. Systematic approaches, standard operating protocol adherence and (repeated) training across sites may be of assistance [102, 103]. In addition, large-scaled retrospective studies of electronic medical records (EMRs) (e.g. Sun et al., [67]), and correlating biomarkers or biomarker profiles with disease-progression predictions [76], is recommended.

The studies assessed in this review highlight the presence of peripheral inflammation in ALS, and more specifically, the differential monocytic gene expression profile both compared to controls and correlated to disease severity. Further interrogation of this cell population and its response to ALS-relevant stressors in vitro, by combining soluble factor analysis, proteomics, transcriptomic analysis (RNA, miRNA, circRNA), and machine-learning approaches may be particularly fruitful for the identification of novel biomarkers, or biomarker profiles, in ALS.

## Data Availability

Not applicable.

## Abbreviations

AALS: Appel ALS score
ALS: Amyotrophic Lateral Sclerosis
ALSFRS-R: ALS Functional Rating Scale-Revised
ASO: Antisense oligonucleotides
AAV: Adeno-associated virus
BEST: Biomarker EndpointS and other Tools
CCL2: C-C Motif Chemokine Ligand 2
CMAP: Compound motor action potential
CNS: Central nervous system
CSF: Cerebrospinal fluid
CRP: C-reactive protein
CXCL1: C-X-C Motif Chemokine Ligand 1
DTI: Diffusion tensor imaging
EMRs: Electronic Medical Records
EU: European Union
FDA: Food and Drug Administration
FVC: Forced Vital Capacity
IFNγ: Interferon gamma
IL: Interleukin
MCP-1: Monocyte chemoattractant protein 1
MG: Myasthenia Gravis
miRNA: microRNA
MRI: Magnetic Resonance Imaging
MUNIX: motor unit number index
NfL: Neurofilament light
NK cells: Natural Killer cells
NO: Nitric oxide
PBMCs: Peripheral blood mononuclear cells
PET: Positron emission tomography
pNfH: Phosphorylated neurofilament heavy
SEB: Staphylococcal enterotoxin B
SOD1: Superoxide Dismutase
TNF: Tumor necrosis factor
